# Response to the Letter to the editor: “cardiovascular magnetic resonance reveals myocardial involvement in patients with active stage of inflammatory bowel disease” (CRCD-D-24–01694)

**DOI:** 10.1007/s00392-025-02600-z

**Published:** 2025-02-05

**Authors:** Maximilian Fenski, Jeanette Schulz-Menger

**Affiliations:** Charite University Hospital Berlin, Charite Universitatsmedizin, Berlin, Germany

Sirs,

We thank Dr. Syeda Rabika and colleagues for their comments on our recently published article [1]. They raised three key points, which we address below.

First, Dr. Rabika and colleagues expressed concerns about the potential distortion of late gadolinium enhancement (LGE) images in patients with cardiac implantable electronic devices (CIEDs). In our study, we used 3D LGE imaging to evaluate left ventricular focal fibrosis and scarring in patients with inflammatory bowel disease (IBD). Notably, none of the participants in our cohort had a CIED. Furthermore, given that left ventricular ejection fraction was preserved across our cohort and no participant had a history of recent ventricular tachycardia, we consider our cohort to be at low risk for future CIED implantation.

Second, Rabika et al. suggested that medical therapy for IBD may reduce the risk of hyperlipidemia and offer cardiovascular protection by downregulating lipogenesis-related genes. While this is an intriguing hypothesis that warrants further investigation, exploring the relationship between IBD medication, lipogenesis-related gene expression, and left ventricular remodeling was beyond the scope of our study. Nonetheless, we agree that prevention, systematic detection, and proactive management of cardiovascular risk factors are essential in IBD management to reduce the risk of cardiovascular events [2].

Third, the authors highlighted that IBD patients may present with a distinct cardiovascular risk profile, including hypertension and hyperlipidemia, compared to the general population. In our study, we found an association between arterial hypertension and the presence of LGE in IBD patients. We hypothesize that the presence of hypertension may modulate the extent of systemic inflammation-induced myocardial damage as indicated by LGE imaging. We agree that future studies should investigate the interplay between systemic inflammatory diseases, obesity, hypertension, diabetes mellitus, and myocardial inflammation.

We appreciate the opportunity to engage in this dialogue and hope these discussions inspire further research in this field.

On behalf of the authors,

Maximilian Fenski and Jeanette Schulz-Menger

## Data Availability

A data availability statement is not applicable, as this letter does not involve new data.
